# Dual-color Colocalization in Single-molecule Localization Microscopy to Determine the Oligomeric State of Proteins in the Plasma Membrane

**DOI:** 10.21769/BioProtoc.4749

**Published:** 2023-07-05

**Authors:** Hua Leonhard Tan, Stefanie Bungert-Plümke, Daniel Kortzak, Christoph Fahlke, Gabriel Stölting

**Affiliations:** 1 Institute of Biological Information Processing, Molecular and Cellular Physiology (IBI-1), Forschungszentrum Jülich, Jülich, Germany; 2 Berlin Institute of Health at Charité – Universitätsmedizin Berlin, Center of Functional Genomics, Hypertension and Molecular Biology of Endocrine Tumors, Berlin, Germany

**Keywords:** Single-molecule localization microscopy, Super resolution microscopy, Quaternary structure, Membrane proteins, PAmCherry, Photoactivated localization microscopy

## Abstract

Determining the oligomeric state of membrane proteins is critical for understanding their function. However, traditional ex situ methods like clear native gel electrophoresis can disrupt protein subunit interactions during sample preparation. In situ methods such as stepwise photobleaching have limitations due to high expression levels and limitations of optical resolution in microscopy. Super-resolution microscopy techniques such as single-molecule localization microscopy (SMLM) have the potential to overcome these limitations, but the stochastic nature of signals can lead to miscounting due to over-expression, background noise, and temporal separation of signals. Additionally, this technique has limited application due to the limited selection of fluorescent labels and the demanding control of laser power. To address these issues, we developed a dual color colocalization (DCC) strategy that offers higher tolerance to background noise and simplifies data acquisition and processing for high-throughput and reliable counting. The DCC strategy was used to determine the oligomeric states of membrane proteins of the SLC17 and SLC26 family with SMLM, providing a robust and efficient method for studying protein interactions.

**Graphical overview**


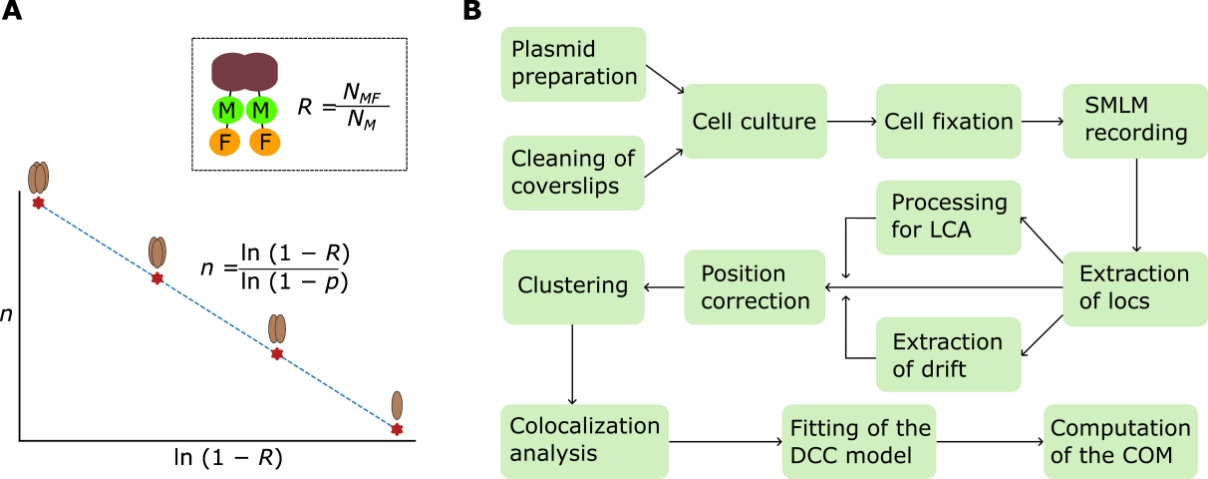


(A) Illustration of the principle for determining the oligomeric state of protein complexes with dual color colocalization–single-molecule localization microscopy (DCC-SMLM). In the inset, as an example, a dimeric protein (brown) is labeled with a marker (M) and an indicator fluorescent protein (F) on each of its two subunits. The overall probability of detecting the dimer with SMLM, as denoted by *R*, the *colocalization ratio*, is equal to the ratio of the number of colocalized marker and indicator clusters (*N_MF_*) to that of the marker clusters (*N_M_*). The plot shows the linear relationship of the oligomeric state (*n*) vs. the natural logarithm of 1 subtracted by the colocalization ratio, supplemented by the equation of the fitting curve, in which *p* denotes the recall rate of the indicator fluorescent protein (F). (B) The workflow diagram shows the procedures of DCC-SMLM (Locs: localizations; COM: coefficient of mismatch; LCA: lateral chromatic aberration).

## Background

The composition of the quaternary structure of a protein tells us how this protein is operating and is therefore critical for the understanding of the molecular mechanisms behind their function. The experimental determination of protein oligomeric states is nontrivial. Several methods or strategies have been developed in the past decades, such as native gel electrophoresis (Schägger and von Jagow, 1991), stepwise photobleaching (Ulbrich and Isacoff, 2007), and single-molecule localization microscopy (SMLM) (Sengupta et al., 2011; Lee et al., 2012). Ex situ methods, such as native gel electrophoresis, rely on the extraction and purification of proteins for further investigation (Schägger and von Jagow, 1991). The detergents and mechanical forces exerted on protein complexes during these steps can, however, result in a loss of weaker interactions between subunits, creating a bias in the observed results. In response to these limitations, in situ methods, primarily centered on fluorescence microscopy, have been increasingly used in the past years. Often, this is in the form of stepwise photobleaching, where protein subunits are labeled with fluorophores and subsequently bleached by strong excitation (Ulbrich and Isacoff, 2007). This results in a stepwise loss of fluorescence for every bleached fluorophore. Due to the spatial limitations of light microscopy, however, this method only works if the density of expressed proteins is low. This mostly limits the application of this method to the use of *Xenopus laevis* oocytes, which are not a faithful representation of the cytosolic environment in mammalian cells.

As a rather elegant strategy to implement super resolution microscopy technique, SMLM temporally separates the emissions from individual molecules to avoid overlapping signals from multiple emitters in the same diffraction limited spot. Fitting of these isolated signals can increase the resolution by up to tenfold. To achieve homogeneous labeling, the fluorophores are usually genetically encoded photoactivatable fluorescent proteins fused to the protein of interest. Thus, this type of SMLM is also called photoactivated localization microscopy (Betzig et al., 2006).

Despite the achievable resolution and genetic labeling, it is still not possible to count the number of proteins within a sample by the number of fluorescent signals observed. For various reasons, not all fluorescent proteins will generate a signal during experiments (i.e., the recall rate is lower than 1). The distribution of the number of counts per protein complex is described by a binomial distribution, defined by the recall rate (Nan et al., 2013; Durisic et al., 2014). This strategy faces numerous challenges, primarily in that it demands a high recall rate (at least 0.5) of the fluorescent proteins. There are only a few options of suitable fluorescent proteins reported previously [e.g., PAmCherry (Durisic et al., 2014) and mEos3.2 (Zhang et al., 2012)]. Emission events from each fluorescent protein can extend over several frames and may be discontinuous. It is therefore imperative to develop algorithms that assign emission events to individual proteins within the complex. Furthermore, the binomial distributions of signals are often spuriously affected by a significant amount of background signals that are almost indistinguishable from true signals. We recently published a novel strategy, dual color colocalization (DCC) (Tan et al., 2022), circumventing these problems, as illustrated in the Graphical overview. The DCC strategy uses two covalently linked fluorescent proteins, serving as the marker (M) and the indicator (F), fused to the protein of interest. The marker is used to select the fraction of protein complexes that will be used for counting, while the indicator will be used to determine the oligomeric state (*n*) of the protein complex. This is done by experimental determination of the detection probability (*R*) of the protein complex, as given by the colocalization of the two fluorescent proteins:

$R=N_{MF}\text{/}N_{M}=1-{\left(1-p\right)}^{n}$ (Equation 1)

*N_MF_* is the number of protein complexes detected via both M and F, i.e., the colocalized protein complexes. *N_M_* is the overall number of clusters that show fluorescence of M, regardless of whether they are colocalized with F or not, and *p* is the recall rate of the indicator F. In this way, we disregard the complicated temporal separation (except to improve resolution based on SMLM) and assignment of emission events to individual proteins. This simplifies the imaging procedure and the data processing and builds a direct connection between the detection probability of the protein complex and its oligomeric state (Equation 1).

Since our method relies on a cumulative probability instead of the probability densities of multiple detected states in a binomial distribution, the DCC strategy does not demand a high recall rate, thereby broadening the spectrum of usable fluorophores. For the marker protein, we choose the bright mVenus, given that the corresponding filter setting leads to a very low noise level, which is essential for the faithfulness of the result.

Overall, this dual-color strategy greatly increases the signal-to-noise ratio in comparison with the single-color counting strategy. As we considered that the two adjacently linked fluorescent proteins may interfere with one another, or be truncated simultaneously, we elaborated our model by introducing another parameter, the coefficient of modification denoted by *m*, resulting in the following formula:

R=∑k=1nnk1-mkmn-k1-mn1-1-pk (Equation 2)

In this protocol, we present the best practices that we established during the development of our method. We used DCC to determine the in situ oligomeric states of the vGlut family as monomers and SLC26 family as dimers. The DCC strategy has been proven to be an efficient improvement of the conventional strategy using SMLM to determine protein oligomeric state.

## Materials and reagents

1. Filter for solutions, 0.22 μm (e.g., Corning^®^ 1,000 mL Bottle Top Vacuum Filter, catalog number: 431174)
2. Sterile cell culture Petri dishes [Sigma-Aldrich, catalog number: D7804 (35 mm) and catalog number: Z755923 (100 mm)]
3. Low background coverslips, 25 mm, No. 1 (VWR, catalog number: 631-1584)
4. Microscope slides, 75 mm × 25 mm, plain (VWR, catalog number: 48300-026)
5. HEK293T cells (Sigma-Aldrich, catalog number: 96121229-1VL)
6. Coplin slide staining jar (glass) for 75 mm × 25 mm slides (DWK Life Sciences, catalog number: UX-48585-20), used for cleaning and storage of coverslips
7. HEK293T cell culture medium made from:
  - DMEM (Thermo Fisher Scientific, catalog number: 10564011)
  - Supplemented with 50 U/mL penicillin-streptomycin (Thermo Fisher Scientific, catalog number: 15140122)
  - Supplemented with 10% fetal bovine serum (FBS) (Thermo Fisher Scientific, catalog number: 16140071)
8. Trypsin-EDTA, 0.05% (Thermo Fisher Scientific, catalog number: 25300062)
9. Lipofectamine 2000 (Life Technologies, catalog number: 11668019)
10. OptiMEM for cell transfection (Thermo Fisher Scientific, catalog number: 31985088)
11. Hydrogen peroxide, 30%, stabilized (Merck, catalog number: 108597)
12. Methanol, gradient grade for liquid chromatography (Merck, catalog number: MX0486)
13. Sulfuric acid, 95%–97% (Sigma-Aldrich, catalog number: 1.00731)
14. Sodium hydroxide (NaOH) pellets (Merck, catalog number: 106469)
15. Sodium chloride (NaCl) (Sigma-Aldrich, catalog number: S5886)
16. Disodium phosphate (Na_2_HPO_4_) (Sigma-Aldrich, catalog number: S5136)
17. Potassium dihydrogen phosphate (KH_2_PO_4_) (Sigma-Aldrich, catalog number: P5655)
18. Potassium chloride (KCl) (Sigma-Aldrich, catalog number: P5405)
19. TetraSpeck fluorescent bead solution, 100 nm diameter (Thermo Fisher Scientific, catalog number: T7279)
20. pH indicator paper (broad range from acidic to basic; no particular supplier is required)
21. Phosphate-buffered saline (PBS) (see Recipes)

## Equipment

1. Cell culture incubator with a constant temperature at 37 °C and 5% CO_2_
2. Laminar hood with an aspiration pump and a microscope for standard biosafety level 1 cell culture operations
3. Personal safety equipment for handling Piranha solution, including lab coat, acid-resistant gloves, and protective goggles
4. Glassware for preparing buffers and Piranha solution, including 100 mL and 1 L glass beakers, and 10 and 20 mL transfer pipettes.
5. Hot plate with magnetic stirrer for preparing solutions
6. Chemical fume hood for preparing and using Piranha solution
7. Total internal reflection fluorescence (TIRF) microscope capable of recording SMLM images in at least two separate color channels (plus activation 405 nm laser if photoactivatable proteins such as PAmCherry are used). Please consult with your local experts, if necessary, to determine the suitability of any available microscope. As a guide, the microscope we used in our original publication (Tan et al., 2022) was outfitted with the following components (Tang et al., 2015):
  - An ApoN 60× Oil TIRF objective (NA 1.49; Olympus)
  - Three laser lines:
    i. 405 nm diode laser (Cube 405-100C; Coherent) for the activation of PAmCherry
    ii. Argon-ion laser, tuned for 514 nm (Coherent, catalog number: Innova 70C) for imaging of mVenus
    iii. 561 nm diode laser (Coherent, catalog number: Sapphire 561-200 CDRH-CP) for imaging of PAmCherry
  - An acousto-optic tunable filter (AOTF nC-VIS-TN 1001; AA Opto-Electronic, Orsay, France) to control the intensity of the 514 and 561 nm laser beams reaching the objective
  - Two EMCCD cameras (Andor iXon DU897E; Andor, Belfast, UK; one for each color channel) cooled down to -75 °C with liquid nitrogen to record emissions from mVenus and PAmCherry, respectively, using a pixel resolution of 512 × 512 pixels
  - Multi-channel dichroic mirror (e.g., 442/514/561 nm lasers BrightLine^®^ triple-edge laser-flat dichroic beam splitter, catalog number: Di01-R442/514/561-25x36, Semrock)
  - Appropriate emission filters [e.g., 485/537/627 nm BrightLine^®^ triple-band bandpass filter (Semrock, catalog number: FF01-485/537/627-25) and 525/50 nm BrightLine^®^ single-band bandpass filter (Semrock, catalog number: FF03-525/50-25) for mVenus; 609/57 nm BrightLine^®^ single-band bandpass filter (Semrock, catalog number: FF01-609/57-25) for PAmCherry]
8. A recording chamber that holds the coverslip in place without movement during the recording. Furthermore, the chamber must allow for filling with 1–2 mL of imaging buffer (i.e., PBS) on top. (This can be custom-built, or commercially available chambers such as the MS-518 series by ALA Scientific or the 20 series chambers by Warner Instruments can be used.)

## Software

1. Data acquisition system for microscope control and imaging. We used a custom-built program for the original publication that is not available for other microscope systems. In general, however, suitable microscopes come with appropriate software installed. Please consult with your local experts for training in using the microscope and control software, if necessary
2. Fiji (https://imagej.net/ij/download.html)
3. Python 3 with Jupyter Notebook (https://jupyter.org/), Pandas (https://pandas.pydata.org/), NumPy (https://numpy.org/), SciPy (https://scipy.org/), Matplotlib (https://matplotlib.org/), and Pillow (https://pillow.readthedocs.io/en/stable/index.html). To use Python and these packages, we recommend using Anaconda (https://www.anaconda.com/) to manage the packages and solve dependencies
4. SNSMIL (http://english.nanoctr.cas.cn/dai/software/201505/t20150504_146857.html (Tang et al., 2015) or SMAP (https://github.com/jries/SMAP (Ries, 2020) for localization extraction
5. DCC-SMLM library (https://github.com/GabStoelting/DCC-SMLM (Tan et al., 2022)

## Procedure

- **Sample preparation**
  1. Preparation of plasmids
    Clone the cDNA of the proteins of interest (POI) and the standards into the vector plasmid pcDNA3 or pcDNA5/FRT/TO. The proteins must be labeled with two fluorophores—in our case, mVenus and PAmCherry. We chose to fuse the coding sequence of mVenus and PAmCherry to the C-terminus of the coding region of the POI or the protein standard to simplify the procedure and because we had prior knowledge that the function of the proteins would remain largely intact. C-terminal linkage of the fluorophores, however, is not a requirement of our method. Fluorophores may be linked to the N-terminus or embedded within the protein itself. It may be necessary to perform functional experiments to assess whether the function is impaired by linkage to fluorescent proteins such as mVenus and PAmCherry. If possible, it may be possible to link one fluorophore to the N-terminus and the other fluorophore to the C-terminus of the protein. This would eliminate the need to consider unwanted truncation of the fluorophores and thus the need for the parameter *m* (Equation 2).
    We used barttin as the monomeric, the ClC-K and ClC-2 as the dimeric, EAAT2 as the trimeric, and Kcnj2 as the tetrameric standards. However, other plasma membrane proteins can also be tested and used as the standard. Some important criteria for choosing the standard proteins include: (1) the protein should be predominantly in the plasma membrane; (2) the protein should be expressed well in the chosen model cell line; and (3) the protein should have a mostly homogeneous oligomeric state, but not aggregate with others into super complexes or dissociate into individual subunits. We used a linker between the POI or standard protein and the fluorescent protein tag consisting of a flexible hydrophilic sequence with 10–30 amino acid residues. The linker should not be too long since it may increase the chance of protein cleavage. For more details about the standard proteins and the linker sequences, please refer to Tan et al. (2022).
  2. Preparation of coverslips
    - Place the coverslips (25 mm) in the Coplin slide staining jar, standing vertically and separate from each other, so that both sides can be immersed in Piranha solution (see below).
    - Determine the volume of Piranha solution that is needed to completely immerse the coverslips in the Coplin jar. With the Coplin jar and coverslips that we specify in Materials and Reagents, 32 mL is sufficient.
*Note: Piranha solution is hazardous and can be life-threatening if not handled properly! It is advised to consult the safety department of your institution and get training and permission before use. All procedures involving Piranha solution must be performed under a fume hood, and the experimental personnel should wear protective equipment, including lab coat, acid resistant gloves, and goggles. It is very important to keep the area free of any organic solutions, such as acetic acid and ethanol, since mixing Piranha solution with organic solutions is strongly exothermic and may lead to an explosion.*
    - Put a cleaned and dry glass beaker with an acid-resistant stirring bar on a magnetic stirring plate. Do not use heat since preparation of Piranha solution is already strongly exothermic. Starting from this step, the operation must be performed under a chemical fume hood.
    - With a clean glass pipette, add three volumes (3/4 of the total needed volume of Piranha solution) of concentrated sulfuric acid into the beaker. For 32 mL of Piranha solution, three volumes equal 24 mL.
    - Turn on stirring at a low speed, so liquid does not spill out of the beaker.
    - With a glass pipette, slowly add one volume (1/4 of the total needed volume of Piranha solution) of 30% hydrogen peroxide solution to the sulfuric acid in the beaker while slowly stirring it. For 32 mL of Piranha solution, one volume equals 8 mL.
*Note: Heat very quickly builds up and the beaker becomes hot, so use caution when handling it.*
    - Keep the solution stirring for approximately 3 min to fully mix up and stabilize.
    - Pour the Piranha solution into the Coplin jar with coverslips. Before pouring, attach a magnet under the beaker to hold the stirring bar, so it will not fall out.
    - Cover the Coplin jar with the lid but do not seal it since gas will be generated in the cleaning process.
*Note: Sealing the lid can lead to an explosion.*
    - Leave the filled jar under the laminar hood for five days.
    - To handle the residual Piranha solution left in the beaker, use a glass pipette under the laminar hood to slowly add 50 mL of water into the beaker while stirring it on the magnetic plate. While adding water, attach the tip of the glass pipette to the inner wall of the beaker to avoid any splash of liquid out of the beaker.
    - Slowly add 5 M sodium hydroxide solution to neutralize the diluted residue Piranha solution while keeping it stirred. Use a pH indicator paper to confirm neutrality.
    - Pour the neutralized solution into the sink.
    - When the cleaning is done after five days, prepare a large beaker full of crushed ice with a stirring bar on the bottom. The size of this beaker should be at least 30 times the volume of the Piranha solution.
    - Put the beaker on the magnetic plate under the laminar hood. Do not use heat.
    - Carefully pour the Piranha solution onto the ice in the beaker while retaining the coverslips in the Coplin jar.
    - Start stirring slowly.
    - When the ice is melted, carefully add NaOH pellets to the solution to neutralize it while stirring.
    - Discard the solution according to institutional guidelines when it is pH neural, stabilized, and cooled down.
    - Rinse the coverslips in the Coplin jar with double-distilled water 20 times. Collect the waste from the first three rinses into a beaker and neutralize with NaOH solution before discarding.
    - For storage, immerse the cleaned coverslips in double-distilled water in the same jar with the lid sealed with parafilm. Keep refrigerated.
  3. Cell culture and passage of HEK293T cells
    Culture HEK293T cells in culture medium (DMEM supplemented with 50 U/mL penicillin-streptomycin and 10% FBS) in 10 cm Petri dishes at 37 °C in the cell culture incubator supplemented with 5% CO_2_ (Tan et al., 2017). Passage them at least once a week. Refresh the medium every three days. To passage the cells, follow the procedures below:
    - Culture the stock cells with 10 mL of medium in 10 cm Petri dishes. Aspirate the medium from the dish.
    - Wash the cells with 10 mL of PBS three times. Aspirate the solution.
    - Add 1 mL of trypsin solution warmed up to 37 °C in advance to the dish. Evenly spread the solution on the bottom of the dish.
    - Incubate the dish on a 37 °C heating plate for 1 min.
    - Shake the dish on the desk to fully detach the cells from the dish bottom. Check under the cell culture microscope if cells are detached.
    - Add 9 mL of culture medium (pre-warmed to 37 °C) to the dish. Split the cells by resuspending the cell suspension 10 times to get single cells. Examine the cell suspension under the microscope and check if cells are separated.
    - Add 1 mL of cell suspension into a new 10 cm dish with 10 mL of fresh medium pre-warmed to 37 °C. Gently shake the dish to evenly distribute the cells.
    - Place the dish into a cell culture incubator. Passage the cells ten times until replacing with newly thawed cells.
  4. Cell transfection
    - To prepare cells for transfection, use cleaned and sterilized tweezers to transfer a cleaned 25 mm coverslip into a 3.5 cm Petri dish. The coverslips have become hydrophilic during treatment with Piranha solution, so no further surface coating is required for HEK293T cells.
    - Add 2 mL of warm culture medium to the dish.
    - Dispense 0.1 mL of cell suspension onto the coverslip prepared as described above for cell passaging. Swirl the dish carefully to distribute the cells evenly. The volume of the cell suspension should be chosen such that the cells are sparsely distributed on the dish bottom and separate from one another after they settle down.
    - Keep the cells in the incubator for 6 h to let them settle down.
    - Prepare two autoclaved 1.5 mL microcentrifuge tubes and add 100 μL of OptiMEM to each of them. Add 1 μg of plasmid of interest to one tube and 3 μL of Lipofectamine 2000 to the other one.
    - Incubate both tubes separately at room temperature for 5 min.
    - Slowly transfer the DNA-OptiMEM mixture to the Lipofectamine 2000–containing tube drop by drop.
    - Incubate the mixture at room temperature for 20 min.
    - Drop by drop, add the mixture to the 3.5 cm Petri dish of cells prepared 6 h before.
    - Incubate the cells in the incubator overnight.
  5. Cell fixation
    - After the overnight incubation with the transfection mixture, aspirate the culture medium.
    - Wash the cells gently with 2 mL of PBS five times. To avoid introducing impurities, the PBS solution should be filtered with a 0.22 μm sterile filter during preparation, stored in a cleaned and autoclaved glass bottle, and refrigerated.
    - Add 2 mL of -20 °C methanol to the dish and incubate at -20 °C for 5 min.
    - Remove the methanol and wash five times with 2 mL of PBS.
    - Keep the cells in PBS.
- **Imaging of the standard proteins and the proteins of interest**
*Note: The following steps are specific to each individual microscope/control software combination. We give example values and settings for the microscope used in Tang et al. (2015) and Tan et al. (2022). The proper values for your setup (in particular for the camera and laser) will have to be locally determined and adjusted. Please discuss with your appropriate local experts and technicians, if necessary.*
  1. After fixation, take the coverslip with cells from the Petri dish and mount in a recording chamber. Our chamber was custom-made, holding up to 2 mL of solution and being placed on the stage of the microscope, stabilized with magnets.
  2. To track the sample drift and for chromatic aberration corrections, dilute 1 μL of TetraSpeck fluorescent bead solution (vortexed) with 0.5 mL of PBS and then transfer it to the imaging chamber containing the coverslip with the fixed cells.
  3. Leave the sample in a light-proof box for 1 h in the imaging room. This allows the beads to settle and attach to the coverslip (Figure 1A and 1B) and the temperature to reach the lab temperature (22 °C), minimizing sample drift due to thermal shift.
*Note: The lab temperature should be monitored and maintained by air conditioning at a constant temperature (in our case, 22 °C) to avoid thermal drift and to guarantee the optimal performance of the setup.*
    **Figure 1.**
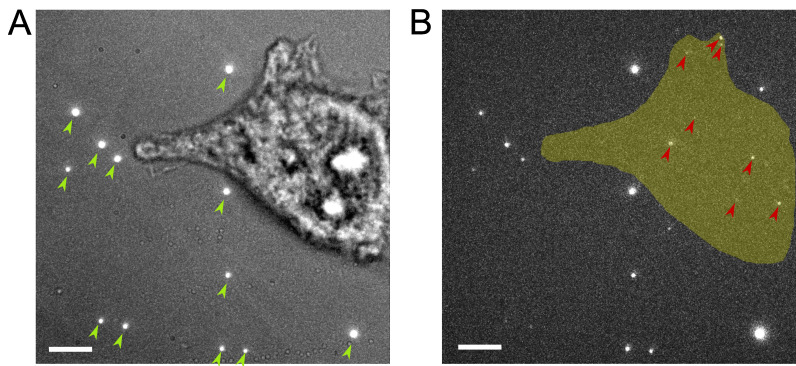
Representative image for the appropriate density of fiducial markers and PAmCherry blinking events. (A) A total of 12 beads (marked by green arrow heads) are attached to the coverslip around the cell after the pre-imaging incubation, shown as bright spots. Cells with beads on top are usually not used for imaging because the bright emissions from the beads interfere with the fluorescent proteins. Scale bar, 5 μm. (B) Gold indicates the area covered by the cell, while the red arrow heads indicate the blinking events recorded within the cell area on the PAmCherry channel during a single frame. Very bright spots around the cell are emissions from the fluorescent beads seen in A. Scale bars, 5 μm.
  4. For the same purpose, add 2 mL of PBS for use as imaging buffer to a 1.5 mL microcentrifuge tube and let it warm up to the lab temperature.
  5. During the bead incubation period, switch on the imaging system to warm it up for at least half an hour.
  6. Align the lasers and adjust the laser intensities at the sample plane according to the manufacturer’s instructions. Laser intensities must be chosen so that individual blinking events are spatially separated to allow for the extraction of super-resolution information. On the other hand, recording durations should allow for the activation and bleaching of all fluorescent proteins present in the sample. As a guide, for our experiments, we measured an intensity of 4.4 mW for the 514 nm laser and 5.4 mW for the 561 nm laser in the sample plane. We increased the intensity of the 405 nm laser during PAmCherry imaging slowly from 3.0 µW to 4.8 mW, so that each frame contained several spatially separated blinking events.
  7. Aspirate the bead solution from the coverslip and rinse it gently with 1 mL of PBS pre-warmed to the lab temperature.
  8. Remove the solution and gently add 1 mL of PBS pre-warmed to the lab temperature.
  9. Put the imaging chamber onto the microscope stage. Let it stabilize for 15 min. To lower the background signal level and therefore increase the signal-to-noise ratio, either darken the lab or, even better, cover the stage area.
  10. Set up the parameters for the camera to have 50 ms exposure and 85.59 ms as the duration between frames, including read-out of data from the camera. The frame duration affects the number of frames that a blinking event covers. Therefore, one must tweak the minimal number of localizations parameter for the clustering analysis if different values are chosen.
  11. In widefield mode, use the green imaging channel (for mVenus) to choose a cell with moderate-to-low expression level of the transfected plasmid.
  12. Move the cell to the center of the view. Around the cell there should be 3–6 beads seen as bright spots in both color channels. Trajectories of the individual beads during the acquisition will be used for the sample drift correction.
  13. In widefield mode, turn on the 561 nm laser for approximately 3,000 frames to bleach impurities within the sample. Background signals will be significantly reduced while PAmCherry molecules are largely unaffected without activation by the 405 nm laser.
  14. Adjust the axial position of the sample stage to bring the beads into focus, as determined by reaching the maximal fluorescence from the beads.
  15. With the white light and the 561 nm laser on, take a transmission image to record the location and shape of the cell and beads.
  16. Switch to TIRF mode and record 4,000 frames with the 514 nm laser for mVenus. Usually, all mVenus molecules should be bleached at the end of recording. In case there are still many blinking events at the end, recording can be continued for another 2,000 frames. If this extended period still does not exhaust blinking events, it indicates either that the expression level is too high, or the laser power is too low. With the emission filter settings and the cleaning procedure, there should be very few blinking events on the green channel from the impurities outside of the cell. This is critical for the DCC algorithm to work.
  17. With the same TIRF settings, record 6,000–12,000 frames with the 561 nm laser for PAmCherry, with 405 nm activation laser intensity increasing from the minimal to the maximal laser power (Section B.4), allowing the emission events to be sparsely distributed (as shown in Figure 1B) and all PAmCherry molecules to be bleached at the end of the recording. If 12,000 frames cannot exhaust all the blinking events within the cell, it may indicate that the expression level is too high.
  18. Adjust the axial position of the sample stage and bring it to focus judging from the intensity of the beads. If the offset is larger than 100 nm, then the recording is considered invalid due to large z-axis drift and must be discarded.
  19. To record beads for the lateral chromatic aberration (LCA) correction using the fitted LCA protocol with the same sample, choose a region on the coverslip that does not contain any transfected cells but only beads (> 10, the more the better as long as they do not overlap with one another). In widefield mode, record 50 frames for each of the green and red channels as a single recording. Slightly move the sample to change the position of the beads and record another 50 frames. Repeat this moving and recording procedure numerous times to extract information from beads in at least 100 different positions, evenly distributed across the whole field of view.
- **Preparation of a bead sample**
  To record beads for the lateral chromatic aberration correction using the regional LCA protocol, prepare a bead-only sample for the SMLM recording. This sample can be stored in the dark at room temperature and lasts for several experiments.
  1. Clean a microscope slide (75 mm × 25 mm) as for the coverslips with Piranha solution and let it air dry before use.
  2. Thoroughly vortex the multi-color TetraSpeck fluorescent bead solution for 1 min.
  3. Add 5 μL of bead solution into 400 μL of double-distilled water in a 1.5 mL Eppendorf tube and then vortex to mix it.
  4. Add the diluted bead solution to a cleaned coverslip (25 mm) and let it air dry at room temperature.
  5. After drying, place the coverslip on a microscope slide with the side of beads facing down towards the slide.
  6. Dip a micropipette tip in nail polish and place 4–5 tiny drops on the edge of the coverslip to glue the coverslip onto the slide.
  7. Once the nail polish is dry, the bead sample is ready for use.
  8. Store the sample in the dark at room temperature until next imaging.
- **Imaging of the bead sample**
*Note: The following steps are specific to each individual microscope/control software combination. The exact values (in particular for the camera and laser) will have to be locally determined and adjusted for your setup. Please discuss with your appropriate local experts and technicians.*
  1. Launch the microscope and align the lasers as for the cell sample.
  2. Mount the bead sample slide on a custom-made chamber that can stick to the microscope stage with magnets. The coverslip should be facing towards the objective when the slide is on the microscope stage.
  3. Place the sample on the microscope stage and let it stabilize for approximately 20 min.
  4. Set up the acquisition parameters so that pixel size, exposure time, and frame rates are the same as those for cell sample imaging. The excitation laser intensities can be reduced to avoid over-exposure since beads are much brighter than fluorescent proteins.
  5. In widefield mode, record the bead sample for 50 frames each in the green and red channels as a single recording.
  6. Slightly move the sample and repeat the imaging if the density of the beads is too low. Usually, a total of 500 beads or 500 positions from fewer beads in the field of view can be sufficient, as shown in Figure 2.
    **Figure 2.**
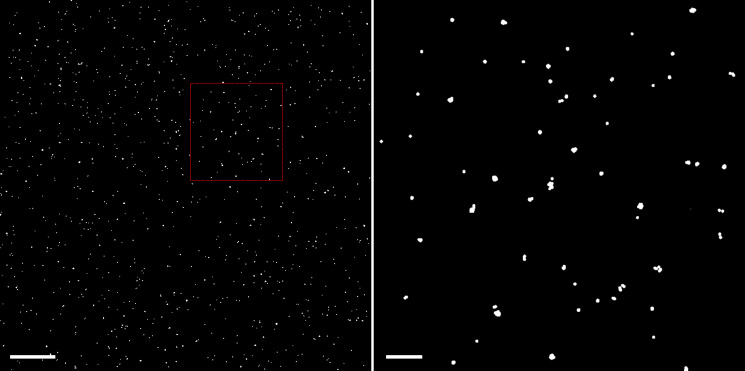
Example of bead recordings used for the lateral chromatic aberration correction. Left: A total of 18 individual bead recordings are combined to occupy the whole camera view (~41 × 41 μm). Each white spot indicates a bead recorded on the PAmCherry channel, spanning 50 frames. Scale bar, 5 μm. Right: Enlarged area defined by the red square in the left image. Scale bars, 1 μm.
- **Extraction of localizations**
  1. Using SNSMIL software:
    We used SNSMIL (Tang et al., 2015) to localize the emission events in the recorded videos (image frames). This software was developed using recordings taken with our microscope setup and is therefore particularly optimized for our use case.
  2. Alternatively, we have used the newer software SMAP (Ries, 2020) to extract localizations from the fluorescence signals in the recorded videos. This software uses improved algorithms and may work better with other microscopes. In our hands, we did not find a general advantage of using either SMAP or SNSMIL.

## Data analysis

**Figure 3.**
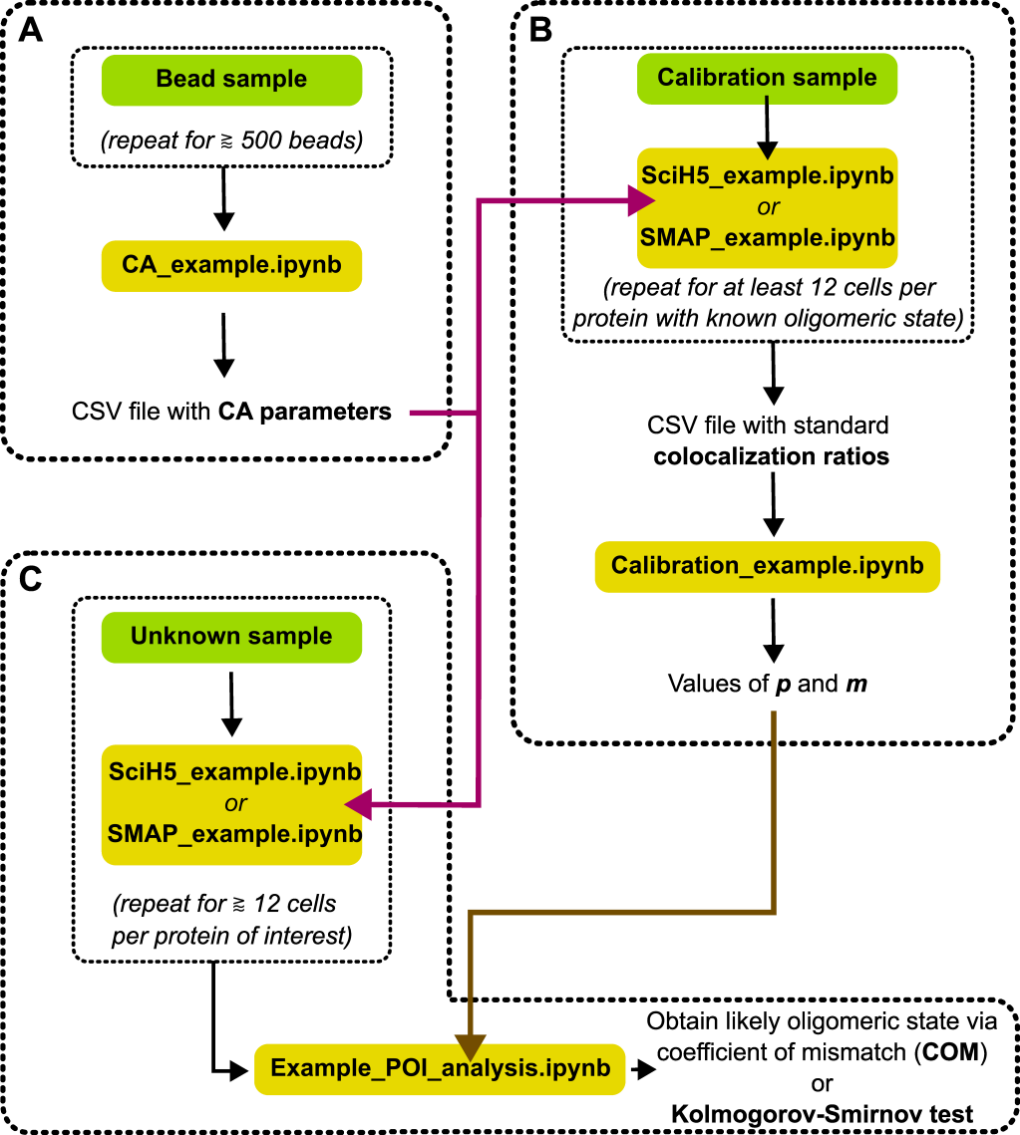
Flowchart for the different steps used during analysis. Green boxes indicate necessary microscope recordings and yellow boxes indicate analysis steps with the mentioned example Jupyter notebook scripts. (A) Two-color recordings of fluorescent TetraSpeck beads are used to determine chromatic aberration (CA). For this, at least 500 beads spread across the whole field of view should be used. It is not required to record 500 separate beads, but the same sample may be moved in between recordings, resulting in multiple recordings that can be stacked together to increase the number of recorded positions. The CA parameters are saved in a .csv file and will be used by the scripts in the other steps (purple arrow). (B) Colocalization ratios of proteins with known oligomeric state must be individually determined. In our experience, at least 12 recordings per protein should be taken. The ratios are then used to calibrate the values of *p* and *m* in the DCC-SMLM model (Equation 2). These values will be used in the next step (brown arrow). (C) Colocalization ratios of proteins with unknown oligomeric states are recorded and individually analyzed. These analyses are then used to determine the most likely oligomeric state of the protein of interest.

The DCC-SMLM algorithms have been integrated into a Python library. To use this library and the example of the analysis as explained below, a working knowledge of the Python programming language is required. For the examples given below, you also need to have some familiarity with Jupyter Notebooks. The protocol below should be studied while simultaneously working with the indicated files as stated in the beginning of each step. We have added a flowchart that may serve as a guide to the necessary steps and files used during analysis (Figure 3).

1. Installation of the DCC-SMLM library
  - Please make sure that you have Python 3 and the Jupyter Notebook installed. If not, we recommend downloading the free version of the Anaconda distribution for your operating system from https://www.anaconda.com. This distribution contains all required tools and libraries to use our analysis scripts.
  - Download our library from https://github.com/GabStoelting/DCC-SMLM into a separate folder. This may be done directly from the GitHub website, using command line, or GUI git tools.
  - If you want to use the library for your own project, we currently recommend copying the DCCSMLM.py file to a new project folder. The example notebooks may be copied and later modified as well.
2. Determination of the chromatic aberration (Figure 3A)
*Note: Open the “CA_example.ipynb” example in Jupyter Notebook and work through the code from top to bottom. The explanations below explain the DCC-SMLM library specific functions as they occur in the Notebook from top to bottom.*
  - Load all extracted localizations from the two-color bead recordings (see Procedure C) in the .SciH5 file from a processed SNSMIL extraction or the .mat output file from SMAP.
  - Identify bead signals using the *find_clusters* function for each color channel. The parameters are:
    i. *intensity_threshold*, which defines the minimum fluorescence intensity per localization in arbitrary units.
    ii. *min_samples*, which sets the minimum number of frames the bead must be recorded in.
    iii. *eps*, which is the epsilon parameter for the underlying DBSCAN clustering, being a measure for the maximal distance between localizations within a cluster.
    iv. *save_column*, which is the name of the column of the internal data structure that will be used to assign each bead a specific ID number.
  - The chromatic aberration between the same bead identified in the green and red color channels is calculated using the *determine_chromatic_aberration* function. In principle, this function identifies the closest bead in one color channel relative to another and measures the deviation. The parameters are:
    i. The first parameter is the bead dataset in the green channel.
    ii. The second parameter is the bead dataset in the red channel.
    iii. The third parameter is the name of the *save_column* set in the previous step.
    iv. Lastly, a cutoff can be specified that will limit the range for the search of beads in the opposite color (*distance_cutoff*).
  - As shown in the literature, chromatic aberration varies linearly relative to the optical point of the microscope. The measured distance of the same bead in each color channel can therefore be plotted as a function of the x- or y-coordinate. A plot of the data from all available beads can be fit with a linear function and used to calculate the necessary correction for every coordinate within the field of view.
  - The fit parameters can be saved and used for correction in other analyses below.
3. Analysis of a single recording (Figure 3B and 3C)
*Note: Open the “SciH5_example.ipynb” or “SMAP_example.ipynb” examples in Jupyter Notebook and work through the code from top to bottom. The explanations below explain the DCC-SMLM library specific functions as they occur in the Notebook from top to bottom.*
  - Load the .SciH5 or .mat file as described above using the *load_channel* function. If you previously determined and saved the parameters of the linear fit for the chromatic aberration correction, you can pass the *ca_file* parameter to that .csv file. This will automatically correct the coordinates of all localizations within the channel.
  - Identify the beads in the recording for determination and correction of sample drift. For this, the *find_clusters* method is again used as described above for the bead sample. Beads can usually be separated by their high fluorescence intensity relative to the signals emitted by fluorescent proteins.
  - The *extract_drift* function will extract the shift of the position of each bead from one frame to the next. Missing data is interpolated as described in our original publication. The mean drift is saved in the *drift* variable of each color channel. The parameters of this function are:
    i. The first parameter contains the localizations of the beads.
    ii. The second parameter contains the name of the column in which the bead IDs were assigned.
  - Clusters of emissions by fluorescent proteins are identified using the *find_clusters* function. The *intensity_threshold* and *min_samples* parameters should typically be much lower than for identifying fluorescent beads.
  - The colocalization ratio can be calculated using the *get_colocalization_ratio* function. The parameters are:
    i. The names of the first color channel.
    ii. The name of the second color channel.
    iii. Name of the bead ID.
    iv. A distance threshold is set using the *distance_cutoff* parameter.
    v. Regions of interest can be defined by providing a list of rectangles in the format (x, y, width, height).
  - The resulting colocalization ratio can be saved for further analysis.
4. Analysis of calibration proteins (Figure 3B)
*Note: Open the “Calibration_example.ipynb” example in Jupyter Notebook and work through the code from top to bottom. The explanations below explain the DCC-SMLM library specific functions as they occur in the Notebook from top to bottom.*
  - Load the file containing the combined colocalization ratios of several recordings of calibration proteins with known oligomeric state as described in the previous step. Use the *DCCReferenceProteins* function for this.
    i. The first parameter is the file name of the .csv file containing the data.
    ii. The second parameter contains the name of the column that contains the colocalization ratios.
    iii. The third defines the name of the column containing the known oligomeric state corresponding to the measured ratio.
  - The *reference_bootstrap* function determines the values of *p* and *m* from the calibration dataset. The first parameter defines the number of bootstraps performed; *save_result = True* will save the values of *p* and *m* to the DCCSMLM object as well as the confidence intervals.
  - The determined values for *m* and *p* can be saved for use in the analysis of proteins with unknown oligomeric state.
5. Estimation of the oligomeric state of proteins of interest (Figure 3C)
*Note: Open the “Example_POI_analysis.ipynb” example in Jupyter Notebook and work through the code from top to bottom. The explanations below explain the DCC-SMLM library specific functions as they occur in the Notebook from top to bottom.*
  - First, load the table with the reference proteins as stated in step 4.
  - Load a file containing the colocalization ratios of several recordings of proteins of interest using the *DCCProteinOfInterest* function. Give the name of the .csv file as first and the name of the column containing the colocalization ratios as second parameter.
  - Now, determine the coefficient of mismatch (COM) as described in our original publication. In summary, the COM is proportional to the difference of the observed colocalization ratio to the expected ratio as calculated from the calibrated values of *m* and *p*. The *com_bootstrap* method calculates the COM for a bootstrapped sample to also give statistical information. The first parameter defines the number of bootstrap samples, and the second parameter contains the object containing the reference data as determined in the previous step. Setting *reference_bootstrap = True* will also bootstrap the reference (calibration) dataset as described above to consider the propagation of the errors of the calibration dataset as well as of the protein of interest.
  - Plot the COM. The smallest value corresponds to the most likely oligomeric state. The bootstrap permutation will also give an estimation of the uncertainty of this value.
  - Additionally, an analysis using a Kolmogorov-Smirnov test can be performed. For this method, the distribution of the colocalization ratios of the protein of interest is compared against the distribution of the calibration proteins. For this, we implemented the *KS* function.

## Recipes

1. **PBS**
  8 g of NaCl, 0.2 g of KCl, 1.15 g of Na_2_HPO_4_·2H_2_O, 0.2 g of KH_2_PO_4_
  Dissolve in 500 mL of double-distilled water and fill up to 1 L. Adjust pH to 7.3–7.4 and filter the solution with the sterile filter. Store in an autoclaved glass bottle at 4 °C.
